# Gandhi and the Decolonisation of Global Health

**DOI:** 10.5334/aogh.4816

**Published:** 2025-09-18

**Authors:** Vikash R. Keshri

**Affiliations:** 1 Jindal School of Public Health and Human Development, O.P. Jindal Global University, Sonipat, Haryana, India; 2 School of Population Health, University of New South Wales, Sydney, Australia

**Keywords:** decolonisation, global health, gandhi, social justice, DEI

## Abstract

The decolonisation of the global health movement has been a matter of intense debate over the last few years. Recent political actions by leaders in the Global North, particularly the closure of the United States Agency for International Development (USAID), call for stronger action by actors in the Global South to strengthen global health. Therefore, this is the right moment for decolonisation in global health to move from rhetoric to action. This essay attempts to inspire action by drawing lessons from the life, struggle and methods of Mahatma Gandhi, who started his life with a Western dream but later led India’s freedom movement through his unique approach of *ahimsa* (non-violence) and *satyagraha* (truth force). Gandhi’s life journey teaches us how decolonisation thoughts develop with the realisation of discrimination and subjugation. His struggles embody how *satyagraha* can be enforced by applying simple means, such as non-cooperation and civil disobedience, and upheld with strict non-violent means. In global health parlance, Gandhi’s concepts can be effectively applied to foster equal and non-subsidiary partnership, based on the principle of *Sarvodaya*—equal opportunity for the most marginalised. His principle of self-reliance must be invoked to build up national capacities. In addition, everyone involved in global health should strive to be ‘the change you want to see in the world’. In the absence of such practice, *satyagraha* should be invoked to ensure fairness in global health.

The decolonisation of global health has been widely discussed and deliberated recently [1]. This is rightly so, as the structure of global health is inherently unequal, often leading to disparities in health outcomes. The feudal nature of global health disproportionately impacts researchers and practitioners in the Global South [2]. However, effective decolonisation requires shifting the movement’s focus from the power of control to the realm of action, which is often situated in the Global South. Unfortunately, many years and several academic exchanges later, the real action for decolonising global health is missing [3]. Here, I propose that lessons from the political decolonisation struggles of the mid-twentieth century, led by many inspiring leaders, can encourage this movement to move ahead.

India’s freedom struggle, spearheaded by Mahatma Gandhi, is a notable example of political decolonisation with non-violent (*ahimsa*) means. Gandhi’s life, struggles and methods offer valuable insights into decolonising global health [4]. The conceptual foundation of Gandhi’s methods can serve as a guiding light for achieving meaningful change in global health decolonisation [5]. Gandhi’s life journey offers critical insights into the natural evolution of decolonisation thoughts and actions. Born into an orthodox Indian family, he initially aspired to a Western dream. He pursued this and became a trained barrister in England. Later, he moved to South Africa to harness promising professional career opportunities [4]. However, he experienced blatant discrimination there that abruptly ended his dreams. In response, he started resistance and called for *satyagraha* (truth force)—a form of nonviolent action that derives strength from adherence to truth [6]. *Satyagraha* relies on two primary strategies: *civil disobedience and non-cooperation. Civil disobedience* entails polite but firm disagreement with practices that perpetuate injustice. *Non-cooperation* involves refusing to participate in or support unjust systems. *Satyagraha* helps fight discrimination and subjugation with non-violence and in the spirit of changing the hearts of stakeholders [6].

After initiating *satyagraha* in South Africa, Gandhi returned to India, where he realised that the level of discrimination and subjugation was much higher. Thereafter, he stayed back and led India’s freedom struggle [5]. Having experienced rights and privileges in the Western world, Gandhi started with similar demands for his fellow countrymen. The learning from Gandhi’s early experiences highlights the evolution of decolonisation thoughts. It starts from awareness of rights and progresses to recognition and realisation of inequality, discrimination and subjugation. This realisation fuels the struggle for equality and calls for a change in the status quo.

Similarly, the decolonisation of global health began with recognising inequities and discrimination within institutions in the Global North. The movement then called for greater diversity, equity and inclusion (DEI) in the northern institutions [3]. Such a call led to fierce debate and called for course corrections in these global north settings. The Global South, which is often the field for global health practice and where subjugation is maximum, remained aloof from such decolonisation movements [7]. However, the recent decision to halt support from the United States Agency for International Development (USAID) and to undermine other institutions funding global health initiatives underscores, more than ever, the urgent need for leadership from the Global South in shaping the future of global health.

Therefore, for the decolonisation in the global health movement to advance meaningfully, the realm of action must shift to the Global South. At present, the dominant voices in the decolonising global health movement remain concentrated among a small circle of elite scholars and practitioners based in the Global North [3]. This must change, and the leadership of the movement must transition to actors in the Global South. The Gandhian methods and examples from his life and struggles offer valuable visions for charting future directions. Here, I outline examples of the future course of action.

First, in any global health collaborations involving research and public health project implementation, when the primary site of work is in the Global South, lead researchers should be based within the region. The majority of the primary funding must also be directed to the institutions in the region. This approach aligns with Gandhi’s vision of freedom, self-rule and global peace. Such efforts must be rooted in genuine collaboration and guided by the spirit of *Vasudhaiva Kutumbakam*—‘the world is one family’—pursued through non-violent means grounded in love, compassion, respect, cooperation and selflessness [4]. In line with the Gandhian principle of trusteeship, funders and institutions in the Global North must treat global health as a global public good. Moreover, Gandhi’s call for *Sarvodaya* (upliftment of all) emphasises equal opportunities for the most marginalised to thrive. Together, these principles should serve as the moral foundation of global health collaborations in the spirit of decolonisation.

Second, aligning with the principle of *self-reliance*, governments, institutions and leaders in the Global South must invest in strengthening their own institutions, developing local capacities and ensuring sustained local financial support to foster long-term self-sufficiency. Furthermore, addressing inequalities in rights and privileges within national boundaries in the Global South is also critical and will require broader institutional reforms. These internal issues should be addressed through national leadership and distinct strategies, separate from the dynamics of the decolonisation movement.

Third, as Gandhi reminded us, ‘Be the change you wish to see in the world’. The responsibility for decolonisation and equality in global health lies with all actors, regardless of their geographic location, institutional affiliation or context. Everyone must commit to practising equality, free of prejudice. However, in the absence of such commitment, the Gandhian principles also offer lessons for raising respectful resistance to achieve decolonisation goals.

Moving ahead, if an institution or actor fails to uphold the principles outlined above, *satyagraha* should be invoked. The first response should be *civil disobedience*—respectful opposition to actions that perpetuate or sustain injustice. If civil disobedience is ineffective, the next course of action is *non-cooperation*—refusal to participate in any global health initiative that lacks fairness and equitable partnerships. Throughout these forms of resistance, truth, *ahimsa* and respectfulness must remain the guiding principles. Moreover, learning from Gandhi’s journey, the champions of decolonising global health should consider relocating to lead such a movement in the actual realm of action.

For the global health decolonisation movement to truly succeed, structural reforms are not optional, but they are critical. The prevailing feudal architecture of global health must be changed through transforming governance and institutional mechanisms. These reforms must be led by the voices and leadership of the Global South. In mapping this path forward, the life and principles of Gandhi offer compelling moral guidance and inspiration for building a just and equitable global health.
